# Choice in episiotomy – fact or fantasy: a qualitative study of women’s experiences of the consent process

**DOI:** 10.1186/s12884-022-04475-8

**Published:** 2022-02-21

**Authors:** Tanya Djanogly, Jacqueline Nicholls, Melissa Whitten, Anne Lanceley

**Affiliations:** 1 UCL Medical School, Medical School Building, University College London, 74 Huntley Street, London, WC1E 6AU UK; 2 Department of Reproductive Health, Faculty of Population Health Sciences, Medical School Building, EGA Institute for Women’s Health, University College London, 74 Huntley Street, London, WC1E 6AU UK; 3 Women’s Health Division, Elizabeth Garrett Anderson Wing, University College London Hospital NHS Foundation Trust, 25 Grafton Way, London, WC1E 6DB UK; 4 Department of Women’s Cancer, Faculty of Population Health Sciences, Medical School Building, EGA Institute for Women’s Health, University College London, 74 Huntley Street, London, WC1E 6AU UK

**Keywords:** Episiotomy, Consent, Patient experience, Qualitative

## Abstract

**Background:**

Consent to episiotomy is subject to the same legal and professional requirements as consent to other interventions, yet is often neglected. This study explores how women experience and perceive the consent process.

**Methods:**

Qualitative research in a large urban teaching hospital in London. Fifteen women who had recently undergone episiotomy were interviewed using a semi-structured interview guide and data was analysed using thematic analysis.

**Results:**

Three themes captured women’s experiences of the episiotomy consent process: 1) Missing information – “We knew what it was, so they didn’t give us details,” 2) Lived experience of contemporaneous, competing events – “There’s no time to think about it,” and 3) Compromised volitional consent – “You have no other option.” Minimal information on episiotomy was shared with participants, particularly concerning risks and alternatives. Practical realities such as time pressure, women’s physical exhaustion and their focus on the baby’s safe delivery, constrained consent discussions. Participants consequently inferred that there was no choice but episiotomy; whilst some women were still happy to agree, others perceived the choice to be illusory and disempowering, and subsequently experienced episiotomy as a distressing event.

**Conclusions:**

Consent to episiotomy is not consistently informed and voluntary and more often takes the form of compliance. Information must be provided to women in a more timely fashion in order to fulfil legal requirements, and to facilitate a sense of genuine choice.

## Background

Episiotomy, a feature of 1 in 7 births in the UK [1], is a surgical incision of the vaginal wall and perineum performed in the second stage of labour. The procedure, used liberally after its introduction in the 1950’s, was thought to prevent severe perineal tearing and long-term pelvic floor damage [2]. However, episiotomy became increasingly controversial as growing evidence demonstrated that its routine use caused worse perineal and vaginal trauma for women [3, 4]. Clinical guidance issued by professional bodies across the globe now mandates the use of episiotomy only in cases of direct clinical need [5–7].

A 2017 Cochrane review evaluating the use of episiotomy, however, noted that trials failed to consider women’s preferences and views on episiotomy, and the outcomes that mattered to them [8]. A World Health Organisation (WHO) recommendation on episiotomy policy suggests that this oversight may extend to episiotomy consent practice: women reported being poorly informed about the procedure and its short- and long-term consequences, and were rarely asked for their consent [9]. The WHO’s global drive for positive birth experiences through woman-centred care and the provision of genuine choice [10] necessitates a clearer understanding of how consent for episiotomy is gained and how women perceive this process.

Despite these reported difficulties, a dearth of resources exists advising clinicians on how to obtain consent for the procedure [11]. Furthermore, consent for episiotomy is often sought at a point when a woman may be exhausted, in pain and unable to fully engage in the consent dialogue. Healthcare professionals are then in an invidious position in which they seek to facilitate consent conversations which accord with their legal and professional duties. A landmark legal case in 2015, *Montgomery *[12]*,* concerning a woman who was not adequately informed about the risks of vaginal birth, established the requirement for clinicians to tailor risk-related and wider information provision to the patient’s circumstances and attendant values. *Montgomery’s* patient-centred test of information disclosure suggests that adequate consent is to some extent defined by the patient – and further, that lawful episiotomy consent practice hinges on an understanding of the values, concerns, and expectations of women who face the procedure.

This study aims to illustrate how consent for episiotomy is currently gained and how women experience this process. This personal and indicative account of consent at childbirth’s most critical moments provides a useful starting point for improvements in obstetric consent practice, which crucially, ought to be guided by patients.

## Methods

### Design

This qualitative study explored women’s experiences of the episiotomy consent encounter using semi-structured interviews. An interpretive and phenomenological methodology was used to inform the collection and analysis of in-depth interview data [13], focusing on the ways in which women constructed meaning into their lived experience of consent to episiotomy.

The data reported is part of a larger exploration of consent processes in different women’s health contexts [14].

### Participants

Women with episiotomies on the postnatal ward at the time of data collection were initially informed of the study by their clinical team. If a woman was interested to hear more about the study, the clinician introduced them to the researcher who provided more detailed written information and answered any questions about the study before inviting the woman to participate and consent in writing, as well as provide demographic details of age, ethnicity, parity and partner, education, and employment status.

Participants were interviewed at a convenient time of their choosing prior to discharge. Participants were sampled consecutively, until no new issues were being raised and data saturation was presumed to have been achieved. This was evidenced during the final interviews and confirmed during initial coding.

### Data collection

Face-to-face, semi-structured interviews were conducted at the woman’s bedside in the postnatal ward by female author TD. To protect a woman’s privacy, women were interviewed at times when the discussions could not be overheard by others and were reminded that the discussion could be paused or halted at any point if they felt self-conscious or, in any way, uncomfortable. Some interviews were short lasting 20 min, whilst others lasted 30–40 min. The researchers were trained in the international Good Clinical Practice (GCP) ethical, scientific and practical standard to which all clinical research is conducted.

Interviews were loosely based on an interview topic guide developed from informal discussions with pregnant women, healthcare professionals and professional staff at a charity concerned with women’s rights in childbirth. The discussion guide (Table 1) supported exploration of the content and perceptions of consent discussions and of women’s overall experience of decision-making.

**Table 1:** Interview topic guide

**What is the purpose of asking for your consent?**
What do you understand by consent?
How does it relate to your view of you making decisions about your care?
How was the purpose of the consent process explained to you?
What do you feel your role was in the process?
**How was the issue of consent for episiotomy raised?**
Who raised the issue of episiotomy?
At what point did these discussions take place?
How were you prepared when you were asked for your consent?
Were you given any preliminary information about episiotomy?
**What were you told when you were asked for your consent to episiotomy?**
What information was given to you in relation to the episiotomy?
Were you given any information sheets, websites, or other sources of information?
Were you asked to sign a consent form?
What do you think are the important things to address when consenting a woman for an episiotomy?
Were risks discussed with you? How were they explained to you?
Were benefits discussed with you? How were they explained to you?
**How did you feel during the consent process?**
What difficulties, if any, did you experience when you were asked for your consent for episiotomy?
Did the doctor check your understanding when seeking consent?
Did you ask any questions?
If so, did you feel satisfied by the answers you received?
Was there anything that you found confusing?
Was it clear to you what you were giving your consent to?
What do you think is the purpose of a consent form?

Interviews were recorded with the permission of the participant and were anonymised and transcribed verbatim by a professional transcription service. Transcripts were double-checked against the recordings to ensure the accuracy and quality of the transcribed data.

Field notes were taken during interviews to contribute to analytic reflection and reflexive research considerations.

### Data analysis

Interview transcripts were analysed using reflexive thematic analysis, based on Braun and Clarke’s six-step inductive method [15]. This involved systematic coding of the entire data set, identifying and grouping interesting features of the data, and generating and refining themes from groups of codes. Coded transcripts and collated themes were checked by and discussed with the research team, with any disagreements resolved through discussion.

## Results

Twenty-one women were approached for the study. Six women declined participation because they were too tired or were to be discharged from hospital shortly. Of the 15 participants who underwent episiotomy, 7 women had forceps delivery, 3 had ventouse and 5 had non-assisted delivery. Fourteen women were primiparous.

Participants are referred to by number (e.g., P1 represents Participant 1). Demographic details, provided in Table 2, are not aligned to individual participants to protect patient confidentiality.

All transcripts demonstrated that consent consultations involve a complex nexus of professional, pragmatic, and individual elements. These were characterised by three themes:

**Table 2:** Participant demographics

	**Number of participants**
**Age**	
20–29	1
30–34	9
35–39	4
40–45	1
**Mean age**	**32.8**
**Ethnicity (self-defined)**	
White British	8
British mixed other	1
White other	1
Mixed	1
Northern European	1
Latin American	1
British Bangladeshi	1
Chinese	1
**Partner status**	
Married	13
Partner	1
Single	1
**Level of education**	
Degree/higher degree	13
Higher education qualification below degree	1
GCSEs	1
**Employment status**	
Full-time	9
Part-time	2
Self-employed	3
Unemployed	1
**Parity**	
First child	14
Second child	1
**Setting of consent**	
Labour ward (doctor-led)	14
Birth centre (midwife-led)	1
**Mode of delivery**	
Non-assisted delivery	5
Forceps	7
Ventouse	3

### Missing information – “We knew what is was, so they didn’t give us details”

Whilst the justifications for carrying out an episiotomy were generally relayed to participants, such as the prevention of tearing (particularly in instrumental delivery) or the speedy delivery of the child, nearly all women reported a lack of discussion on the risks of episiotomy and of being provided with the option to decline. Participants expressed value in understanding the risks and burdens of episiotomy and would have liked to receive this information during consent discussions.

*We didn’t discuss about which other options were on the table, like what would happen if you don’t have the cut […] Then he didn’t mention the risks or side effects […] And since he didn’t mention the risks at all, then of course there was no conversation about the possibility of the risks.* (P11)

*I know what I would have liked to have known […] How it might heal or what might happen if it didn’t heal very well […] In the risk factors on the consent form I signed [for forceps delivery], it didn’t really say anything about [episiotomy]. (P13)*

Brief consent discussions during labour compelled women to rely on any previous knowledge of episiotomy, attained through personal research, antenatal training courses and/or birth plan preparations. Participants resultant opinions on episiotomy were patchy and highlighted the conflicting evidence and controversy that surround it. Yet clinicians too seemed to rely on women’s previous familiarity with the procedure.

*It’s quite a significant intervention that can lead to issues […] Those issues were not really discussed, but I guess I had felt like I had read about those enough and understood them.* (P12)

*[The midwife] didn’t tell you what [episiotomy] was, so if you didn’t know what it was, you wouldn’t know what you were agreeing to […] I said I didn’t want it, or “Can we do anything?” Because I’d done quite a lot of research on tearing and how your body will only tear to what it needs to tear to.* (P8)

### Lived experience of contemporaneous, competing events – “There’s no time to think about it”

The time-critical nature of a birthing context was consistently portrayed as a barrier to proper consent. Some perceived this as an understandable constraint given that consent discussions would detract from the necessary immediate action; others experienced time pressure as a coercive factor that afforded little opportunity for consideration. Many participants felt that it would be both feasible and helpful to discuss episiotomy earlier in the course of labour, as well as before in the form of birth plan discussions, to allow more timely and informed decisions.

*Sometimes you feel forced, if that makes sense, because you’re in a position where you’re scared, you don’t know what – you have to make that decision really quickly; you can’t actually really ponder over it.* (P10)

*If she [the midwife] noticed that things weren’t progressing as well […] she could have said to us, “Look if things don’t carry on like they are in a good way, this is maybe what needs to happen.” And explain the options. Because she is with us a long time, hours.* (P2)

In the context of episiotomy, where the child has somehow to be delivered, reference was made to a societal expectation – said to be held by both mothers and doctors – that the baby ought to take priority. Mothers were often willing to endure any risks on their child’s behalf, leaving no option to refuse an episiotomy.

*In the event that it is the best option for your child, I think that there is no option really*. (P14)

*As I always said, at the end of the day, it just mattered that he [the baby] was okay; I didn’t care [about having an episiotomy]. But I did actually care [through tears], I obviously cared.* (P13)

Many women described the difficulties of decision making given their mental and physical state of exhaustion and pain, which was often experienced as an impediment to genuine consent and compounded by time pressure. Other women, despite the extreme stress on their bodies, defended their capacity to consent.

*I did feel that I had [consented], but at the same time it was all happening, very, very fast and I was probably not very compos mentis, to be honest, mentally, because I was just so tired by that point.* (P12)

*I was tired; it’s another level of tired. But I mean, I definitely still knew that I wanted to say no. (P8)*

### Compromised volitional consent – “You have no other option”

For the majority of participants, episiotomy was presented as a plan of action. They did not feel there was an alternative or that the option of saying no was available to them given the practical constraints described above – still they consented. Reflections on giving consent produced feelings of unease, distress and disempowerment in many participants.

*I said no, and then she said it would be better if she did it, because if I was to tear it could be worse, and I still said no […] I consented in the end, because she was saying the forceps are much bigger […] By that point I’d been in labour for about 40 hours and I was like, err. So yes, I presume I said yes. I think I said yes.* (P8)

*It’s just one of those that even though you are agreeing to a process, sometimes you feel forced […] There’s nothing we can do; I think it’s just the sad situation that you find yourself in.* (P10)

The other half of participants expressed active choice in the sense of placing trust in healthcare professionals and allowing them to make decisions about episiotomy.

*I genuinely trust the doctor’s judgement, I think, yes, I rely on them to make the call on behalf of me in that situation.* (P11)

The voluntariness of consent was influenced by perceived power asymmetries in the doctor-patient relationship, with participants describing feelings of deference when it came to decision making. The sudden appearance of the obstetric team at their delivery sometimes produced feelings of anxiety and vulnerability and left little room for dialogue.

*Obviously, I don’t know everything, so you do have to listen to what they have to say, whether you agree or not, or consent or not.* (P8)

*I was on the table pushing and then suddenly, six or seven of them swarmed in. No one told us why and they just completely took over, and didn’t ask us any questions or any, “Would you be OK with this?” They were more like, “We are going to do this, OK?” And you are so sort of out of it, you just go, “Yeah.”* (P2)

## Discussion

Women do not experience consent for episiotomy as consistently informed and voluntary. Nor do they have a sense of genuine choice. Information provision, particularly regarding the risks of and alternatives to episiotomy, was thought to be inhibited by practical factors including time pressure, concern for the baby’s health and women’s state of exhaustion. Brief discussions around episiotomy, at a point where there was ostensibly no alternative, suggest that the current episiotomy consent process fails to promote a dialogue in which women’s choice-making is prioritised.

This study finds that inadequate information provision on episiotomy, with benefits much more likely to be espoused than risks, severely limits women’s ability to make an informed choice. This is surprising given the evolving legal backdrop in the post-Montgomery era and the ascendant recognition of patient autonomy more generally. Deficiencies in information sharing on episiotomy are evident in other continents: a Brazilian study on women’s perceptions of episiotomy found that half of interviewees received no information about episiotomy before or during childbirth and were particularly unaware of its consequences [16], whilst a qualitative study in China similarly identified a lack of advanced information on the procedure [17]. The ubiquity of poor consent taking for episiotomy suggests that a reappraisal of the current consent process is required.

A lack of time was consistently perceived by participants as a barrier to information provision. Certainly, many maternal negligence cases relate to ‘failure to intervene’ or ‘delay in intervention,’ [18] meaning that doctors may be practising in rapidly changing circumstances. Indeed, participants mainly recounted how choice was removed as the labour progressed and problems arose, a pattern which has been observed in other qualitative studies [19]. We suggest that information on episiotomy could have been shared during the many hours that participants were on the wards, in which time they experienced several other productive consent encounters. This is particularly pertinent when the likelihood of episiotomy is high, such as in first-time mothers [20], which 14 of 15 study participants were, or with assisted delivery [21], which two thirds of participants underwent.

Even so, the assumption that episiotomy is a likely component of assisted delivery is challenged by recently updated clinical guidance in the UK. Reflecting the legal guidance in *Montgomery*, the Royal College of Obstetricians and Gynaecologists concludes that there is insufficient evidence to support either routine or restrictive use of episiotomy in operative vaginal delivery, and that decisions should therefore ‘be tailored to the circumstances at the time and the woman’s preferences’ [22]. These critical discussions may influence a woman’s choice between instrumental delivery and caesarean-section, with a growing body of evidence suggesting that the likelihood of pelvic injury should be canvassed in such risk–benefit equations [23]. That women in this study did not experience such tailored consent consultations is concerning and presumably reflects the challenge inherent in the legal and professional requirements.

Several factors also challenged the voluntariness of participants’ consent. A sense of genuine choice was particularly undermined by a birthing context in which the interests of the baby are absolute. This attitude was condemned in the *Montgomery* ruling:

In this day and age, we are not only concerned about risks to the baby. We are equally, if not more, concerned about risks to the mother. And those include the risks associated with giving birth, as well as any after-effects [14].

Previous research demonstrates the pervasiveness of perineal pain and discomfort following episiotomy and its social, psychological, and sexual impact [24–27]. Yet outcomes for the child are regarded as a key measure of women’s satisfaction with their birth experience [28], and in the face of concern for the child, participants were largely compliant with the loss of choice. Once a healthy child was born, women were perhaps less likely to criticise the consent process or admit dissatisfaction with their birth experience. Our findings do not indicate that women would necessarily have made a different choice, rather they suggest that there is a need for consent consultations to be more sensitively handled such that women are supported in making balanced decisions which acknowledge and validate their interests and concerns – which may be separate from those of the child. In this study we did not re-interview women after discharge, but it seems plausible that a more sensitive consent process may help women to cope with the legacy of an episiotomy.

Participants also alluded to power asymmetries in the doctor-patient relationship that affected their compliance and were sometimes perceived as coercive. Episiotomy was proposed by clinicians with surety and minus other options, creating conditions in which a woman’s reluctance to challenge a doctor’s recommendation is reinforced [29]. Undue reliance by doctors on the patient to voice their concerns, particularly given the vulnerable state that participants described themselves to be in, disregards the realities of an ‘inherent power dynamic’ in maternity care [30], and may interfere with a patient’s legal right to make an autonomous decision. Whilst total compliance with a doctor’s recommendations is a voluntary and understandable decision, common amongst pregnant women [31], other options must be presented in order to prevent choice from being merely an ‘illusion’ in maternity care, as previous authors have cautioned [32]. We have data to suggest that some women felt belittled, but issues of abuse or obstetric violence were not the focus of this study. The issue merits a separate study to facilitate more detailed exploration which we plan to undertake*.*

Further to the selective disclosure on episiotomy, in both timing and information, was the under-utilisation of women’s birth plans. Beyond empowering and engaging women in their care [33], birth plans could helpfully guide consent discussions and the tailoring of information provision to the individual, a task that clinicians have justifiably identified the difficulty of achieving in a limited timeframe [34]. Participants described presenting at labour with highly personalised and considered birth plans, many of which clarified their stance on episiotomy, but which were not necessarily heeded by healthcare professionals. These should be employed to facilitate a collaborative exchange of information at the appropriate time, in the process dismantling power structures and ensuring that women’s voices are heard and listened to. This need has been recognised by a consortium of professional colleagues who are working to develop a digital tool, for use by healthcare professionals and women and their partners during childbirth, that aids the woman in making an informed decision about next steps during labour; our findings reiterate the pressing need for such initiatives [35].

We believe this is the first study to explore women’s experiences of consent for recent episiotomy in England. Key strengths of this study were the recency of women’s experiences and the researcher’s immersion in the clinical environment such that interviews were able to be conducted in the relative informality of the participant’s bedside, which facilitated women’s unguarded reflections on their experience. Whilst difficulties recalling accurate information were minimised by conducting interviews shortly after birth, it would be interesting to interview women at a later date to see if their views changed once their episiotomy has healed. In addition, ethnographic observations of the episiotomy consent process in its clinical setting would provide valuable contextual understanding. Although our study uncovered many key issues all participants had a good command of English and had given birth in the same care facility so it is unknown whether our findings would be replicated in non-English speaking women and/or women in different clinical sites. Whether the results have relevance to other antenatal care services and to other types of consent decision-making remains open although responses to this paper may strengthen such generalisability.

## Conclusion

Genuine choice must be offered to women through the provision of information on the risks, aftereffects, and alternatives to episiotomy, with adequate opportunity for consideration. Women’s birth plans present a valuable springboard for discussion before and during labour. Such steps will ensure that consent to episiotomy is both informed and voluntary, fulfilling legal and professional requirements with mutual benefit for both doctor and patient.

## Data Availability

The fully anonymised datasets used and/or analysed during the current study are available from the corresponding author on reasonable request from authenticated researchers. The study was conducted by HEFCE funded researchers.
